# New population of *Solanum pimpinellifolium* backcross inbred lines as a resource for heat stress tolerance in tomato

**DOI:** 10.3389/fpls.2024.1386824

**Published:** 2024-07-01

**Authors:** Neta Bashary, Golan Miller, Tzion Doitsch-Movshovits, Avital Beery, Bo Ouyang, Michal Lieberman-Lazarovich

**Affiliations:** ^1^ Department of Vegetables and Field Crops Sciences, Institute of Plant Sciences, Agricultural Research Organization - Volcani Center, Rishon LeZion, Israel; ^2^ National Key Laboratory for Germplasm Innovation and Utilization of Horticultural Crops, College of Horticulture and Forestry Sciences, Huazhong Agricultural University, Wuhan, Hubei, China

**Keywords:** tomato, moderate chronic heat stress, fruit-set, pollen viability, *Cpn60*

## Abstract

The occurring temperature increase in crop production areas worldwide is generating conditions of heat stress that negatively affect crop productivity. Tomato (*Solanum lycopersicum*), a major vegetable crop, is highly susceptible to elevated temperatures. Under such conditions, fruit set is dramatically reduced, leading to significant yield losses. *Solanum pimpinellifolium*, a wild species closely related to the cultivated tomato, was shown to have beneficial attributes under various abiotic stress growth conditions. We have utilized a new population of backcross inbred lines originated from a cross between *S. pimpinellifolium* and *S. lycopersicum*, in order to evaluate its potential as a new genetic resource for improvement of reproductive performance of cultivated tomato under heat stress conditions. This population was screened for various heat stress-related traits, under controlled heat stress and non-stress conditions. Our results show that significant variation exists for all the heat stress related traits that were examined and point at individual lines with better reproductive performance under heat stress conditions that share a common introgression from the wild *S. pimpinellifolium* parent, suggesting several candidate genes as potential drivers of thermotolerance. Thus, our results place this population as a valuable new resource for the discovery of heat stress related genetic loci for the future development of heat stress tolerant tomato cultivars.

## 1 Introduction

Tomato (*Solanum lycopersicum*) is one of the most important vegetable crops worldwide. Similarly to other plant species, tomato is very sensitive to high ambient temperatures, a globally increasing phenomenon due to climatic changes. For tomato plants, temperatures exceeding 32°C during the day, and 21°C during the night create conditions of heat stress, leading to various physiological and developmental effects that drive dramatic reduction in fruit set and total yield, thus necessitating the identification of novel resources for tomato breeding (Hazra et al., 2007; Liu et al., 2023). The negative effect of heat stress on yield is the consequence of multiple molecular, physiological and developmental processes that are hindered by elevated temperatures, underlining tomato thermotolerance a highly complex trait.

The reproductive phase of development is more thermosensitive compared with the vegetative phase. Starting at early stages of flowering, the number of flowers produced under high temperature conditions may be reduced (Charles and Harris, 1972; Abdul-Baki, 1991; Xu et al., 2017). Additionally, flower abnormalities are very abundant under heat stress conditions, therefore these traits are indicative of heat stress sensing and damage. High temperatures may also lead to protrusion of the style over the anther cone thus hindering self-pollination (Rick and Dempsey, 1969; Lohar and Peat, 1998). Excessive heat stress may also result in anther deformations, as the cone-shaped anthers burst open, hindering pollination as well (Bita and Gerats, 2013; Müller et al., 2016; Ayenan et al., 2019). The most dramatic effect of heat stress on tomato flowers is bud abscission (Sato et al., 2001). Nevertheless, the most sensitive tissue to elevated temperatures is the male gametophyte in the period of 8–13 days prior to anthesis (Sato et al., 2002). High temperatures lead to reduced pollen viability and germination, vital functions for successful fertilization (Charles and Harris, 1972; Peet et al., 1998; Pressman et al., 2002). Consequently, fruit set is severely damaged. For example, under mean daily temperature of 29°C compared with control conditions at 25°C, tomato fruit set was reduced by 77% (Peet et al., 1997). Later studies presented similar results under different heat stress regimes (Sato et al., 2000; Firon et al., 2006; Sato et al., 2006). Due to the reduced ability of the pollen grains to initiate fertilization under heat stress conditions, the number of seeds per fruit is significantly reduced, occasionally accompanied by a reduction in fruit size (Sato et al., 2001).

For most crop species, the largest reservoir of genetic variation exists not within modern cultivars, but in their undomesticated wild relatives. In tomato, it was estimated that less than 5% of the available genetic variation exists in cultivars and the other 95% is found in wild relatives (Miller and Tanksley, 1990). For this reason, interrogating wild relatives for abiotic stress resistance traits is of great value. To-date, 17 species of wild tomato relatives are recognized, originating from various environments, from high mountains to dry coastal regions, and they exhibit great differences in morphological characters, disease susceptibility, and stress resistance traits (Kimura and Sinha, 2008; Peralta et al., 2008; Conesa et al., 2017). The use of wild relatives may be sometimes hindered due to cross incompatibility, F1 hybrid sterility, infertility of the segregating generations, reduced recombination between the chromosomes of the two species, or linkage-drag (genes of negative effect being tightly linked to the trait of interest) (Zamir, 2001). Despite these obstacles, backcross breeding was successfully used in tomato to improve quantitative traits by the introgression of genes from wild germplasm into elite cultivars while maintaining the favorable horticultural characteristics of the elite materials (Hartman and St Clair, 1999; Doganlar et al., 2002; Kabelka et al., 2004).

The wild species *Solanum pimpinellifolium*, considered as the ancestor of the cultivated tomato, is originated in the coastal deserts of Peru and Ecuador (Peralta and Spooner, 2000; Pease et al., 2016). The *S. lycopersicum* and *S. pimpinellifolium* species are both self-compatible, red-fruited and they are known to hybridize naturally (Rick, 1958). Despite their close relationship, the two species differ in many morphological aspects, especially in fruit size and growth habits and several other economically important traits, many of which are polygenic. For this reason, *S. pimpinellifolium* is considered an attractive source for tomato breeding. Several studies have already utilized *S. pimpinellifolium* for the identification of important quantitative trait loci (QTLs). Among these are QTLs for increased yield, soluble solids content, and improved fruit color (Grandillo and Tanksley, 1996). *S. pimpinellifolium* was shown to be a suitable source for improving nitrogen use efficiency. A collection of 29 Introgression Lines (ILs) resulting from a cross between the To-937 accession of *S. pimpinellifolium* and the *S. lycopersicum* cv Moneymaker (MM) was used to identify specific regions in the *S. pimpinellifolium* genome involved in the responses to N inputs of fruit production and fruit quality. Interestingly, the identified region contains genes involved in C and N metabolism (Renau-Morata et al., 2024).

In terms of abiotic stress tolerance, it was reported that seeds of *S. pimpinellifolium* exhibit better germination under drought stress compared with cultivated tomato (Lin et al., 2002; Villalta et al., 2008; Rao et al., 2013), while five QTLs were found to be linked with salt tolerance during vegetative growth (Foolad et al., 2001). Additionally, several *S. pimpinellifolium* accessions were characterized as having high salinity tolerance (Lin et al., 2002; Villalta et al., 2008; Rao et al., 2013). In regard to heat stress, pollen number, pollen viability and style protrusion, representing key reproductive heat stress-related traits, were evaluated in various wild species, among them eight accessions of *S. pimpinellifolium* (LA1670, LA1630, LA1645, LA1629, LA0114, LA1579, LA1237 and LA1547). Although no overall thermotolerant species was identified, several *S. pimpinellifolium* individuals of the LA1630 accession outperformed the best performing cultivars in terms of pollen viability under heat stress conditions (Driedonks et al., 2018). In another study, 22 QTLs involved in reproductive traits at different temperatures were identified, using an *S. pimpinellifolium* recombinant inbred lines (RIL) population. All 168 lines were subjected to increased temperature conditions and individual lines presented improved performance under these conditions (Gonzalo et al., 2020), demonstrating the high value and potential of *S. pimpinellifolium* as a genetic source for heat stress tolerance in tomato.

Climatic models in tomato-growing locations around the globe predict that temperatures will continue to rise and the severity and frequency of above-optimal temperature episodes will increase (Bell et al., 2000). These conditions strongly and negatively affect reproductive success and thus yield, implying that breeding for thermotolerant cultivars is critical for food security. The sub-optimal growth conditions in which *S. pimpinellifolium* was evolved in, its proven resistance to various abiotic stresses and its genetic relatedness to the cultivated species *S. lycopersicum*, place it as a good source for the identification of heat stress tolerance traits. Here we present a detailed phenotypic characterization of heat stress related traits of a new population based on *S. pimpinellifolium*. Using this new BILs (Backcross Introgression Lines) population originated from a cross between *S. pimpinellifolium* and *S. lycopersicum*, we found high variation in heat stress related traits, and identified specific lines with better performance under heat stress conditions, making this BILs population an additional and highly relevant source for thermotolerance QTL identification and breeding of thermotolerant tomato cultivars.

## 2 Materials and methods

### 2.1 Plant material

The BILs population was obtained from the laboratory of Prof. Dani Zamir (The Robert H. Smith Faculty of Agriculture, Food and Environment at the Hebrew University of Jerusalem). This population was constructed by crossing *S. lycopersicum* cv. M82 and the wild specie *S. pimpinellifolium* accession LA1589. Two backcross cycles to *S. lycopersicum* TA209 and nine generations of self-fertilization resulted in a set of 166 homozygous BC2S9 lines (M82 and TA209 being Israeli and US adapted open-pollinated varieties, respectively, and are almost identical at the genome sequence level).

### 2.2 Growth conditions

All three experiments were carried out at the Agricultural Research Organization (ARO) Volcani Center greenhouses. The plants were planted in 10 L pots filled with a soil mixture (GREEN, EvenAri LTD, Israel) and fertilized with Gat fertilizer (Deshen Gat LTD, Israel) containing M6% + Ca1.5% + Mg0.9%. In the non-controlled experiment, the entire BILs population (166 lines) along with the M82 and *S. pimpinellifolium* LA1589 parental lines were grown in a greenhouse between June and December 2018, therefore experiencing high ambient temperatures. Temperature was monitored every ten minutes using HOBO data loggers (U-Series Data Logger, Onset Computer Corporation, USA). The average daily temperature was 30/24°C day/night. The average maximum temperature during the day was 36°C and the minimum at night was 21°C, creating Moderate Chronic Heat Stress (MCHS) conditions. Under these conditions, plants were scored for various developmental and heat stress related traits. Next, in the controlled experiment (repeated twice), due to space limitations, 14 lines that represent the phenotypic variation in this population and include 90.12%of the *S. pimpinellifolium* genome (**Supplementary Figure S2**) were grown together with the parental lines. The experiment was carried out in two controlled greenhouses and included four plants per line in each greenhouse, in a randomized set up, identical between the greenhouses. First, both greenhouses were set at the same, non-stress temperatures (26°C/20°C day/night – control conditions). At the onset of flowering, MCHS was initiated in one of the greenhouses, by setting day/night temperatures to 32°C/22°C. The other greenhouse was kept at control conditions (26°C/20°C day/night). Temperature was recorded every 15 minutes using HOBO data loggers (U-Series Data Logger, Onset Computer Corporation, USA). Two weeks after the initiation of MCHS, three inflorescences per plant were marked and scored for the different traits.

### 2.3 Phenotypic analyses

#### 2.3.1 Flower types

Three inflorescences were marked per plant, and flowers were scored as: normal, style elongated, anthers deformed, the appearance of anther browning, or aborted. Percentage per type per inflorescence was calculated and averaged per line.

#### 2.3.2 Number of fruits and fruit set

The number of fruits from three to four inflorescences per plant were counted and averaged. Per inflorescence, the percentage of fruits out of total flowers was calculated to obtain the percentage of fruit set.

#### 2.3.3 Fruit weight

All fruits from the marked inflorescences were collected at the red-ripe stage and weighted. Single fruit weight was calculated by dividing the total weight in the number of fruits, per plant.

#### 2.3.4 Pollen viability

Three flowers at anthesis per plant were collected (at early morning) and anthers separated. Each anther was divided in two and inserted into a tube containing germination solution (Sato et al., 2000), followed by adding 20 μL of Alexander dye (Alexander, 1980). Samples were analyzed using a Leica DMLB epi-fluorescence microscope (Leica, Germany). Three fields containing representative pollen patterns were captured with a DS-Fi1 digital camera using NIS-Elements BR3.0 software (Nikon, Japan). Viable (purple) and non-viable (blue/green) pollen grains were counted with the ImageJ software version 1.43 using the ‘Cell counter’ plugin (Schneider et al., 2012).

#### 2.3.5 Pollen germination

Three flowers at anthesis from each plant were collected and dried for 1 h. Dried anthers were transferred to 0.5 mL of liquid germination media (20 mg H_3_BO_3_, 60 mg CaNO_3_, 40 mg MgSO_4_, 20 mg KNO_3_, 10 g sucrose, 100 mL double distilled water) and vortexed vigorously for 10 s. SeaKem^®^ LE Agarose (LONZA Company) was added to the liquid media (to a final concentration of 2%), dissolved and poured onto a microscope slide, flatted with a Parafilm^®^ tape and another slide on top. After solidification, slides were transferred to a dark closed chamber to maintain humidity. Then, the pollen containing solution was transferred to the solid media and incubated for 1.5 h. Slides were analyzed using a DM500 Leica microscope (Leica, Germany). Pollen germination was scored and counted using the ImageJ software version 1.43 (Schneider et al., 2012).

#### 2.3.6 Number of seeds

Tomato red ripe fruits were cut horizontally and all seeds were extracted and incubated with Sulfuric acid (2%) for 3 h. Then, the seeds were washed well in tap water and sterilized with a 10% TSP (Tri Sodium Phosphate) solution for 30 minutes while shaking. Seeds were washed, dried, and counted by picture analysis using ImageJ (Schneider et al., 2012).

### 2.4 Statistical analyses

The Students’ t-test was employed to identify significant differences between lines (p < 0.05). All statistical analyses were performed using JMP Version 3.2.2 (SAS Institute, Inc., Cary, NC, USA).

### 2.5 RNA isolation and quantitative-real-time PCR analysis

Young leaves from three plants per line were collected and frozen immediately in liquid nitrogen. Total RNA was isolated using RiboEx™ (GeneAll; Seoul, South Korea) according to the manufacturer’s protocol. RNA was treated with DNaseI (Thermo Fischer Scientific, CA, USA) and cDNA was synthesized from 0.5 μg of DNaseI-treated total RNA using the qPCRBIO cDNA Synthesis Kit (PCR Biosystems, London, UK) according to the manufacturer’s instructions. Relative expression of *Hsp17.6* (NM_001246984.3) was determined using quantitative real-time PCR (qRT-PCR) on an Applied Biosystems StepOnePlus Real-Time PCR System (Thermo Fischer Scientific, CA, USA). *Ubiquitin* (*Solyc07g064130*) and *EF1α* (*Solyc06g009970*) were used as reference genes. Each 10 μl qRT-PCR reaction consisted of qPCRBIO SyGreen Blue Mix Hi-ROX (PCR Biosystems, London, UK), gene-specific primers, and 1:5 diluted cDNA template. Thermocycling conditions were performed according to the manufacturer’s instructions. Data were analyzed using the Applied Biosystems StepOne™ software v 2.3 (Thermo Fischer Scientific, CA, USA). Primer sequences are as follows (all 5’ to 3’); *Hsp17.6* (*Solyc08g062450*) forward: GGAAGAGGGAAGAAGAGAAAGAA, reverse: ACCACAAACCATCAAAACAGAGT. Ubiquitin reference gene (*Solyc07g064130*) forward: GGACGGACGTACTCTAGCTGAT, reverse: AGCTTTCGACCTCAAGGGTA. For *eEF1α* (*Solyc06g009970*), forward: AGTCAACTACCACTGGTCAC, reverse: GTGCAGTAGTACTTAGTGGTC.

## 3 Results

### 3.1 Developmental variation in the *S. pimpinellifolium* BILs population

**Figure 1:**
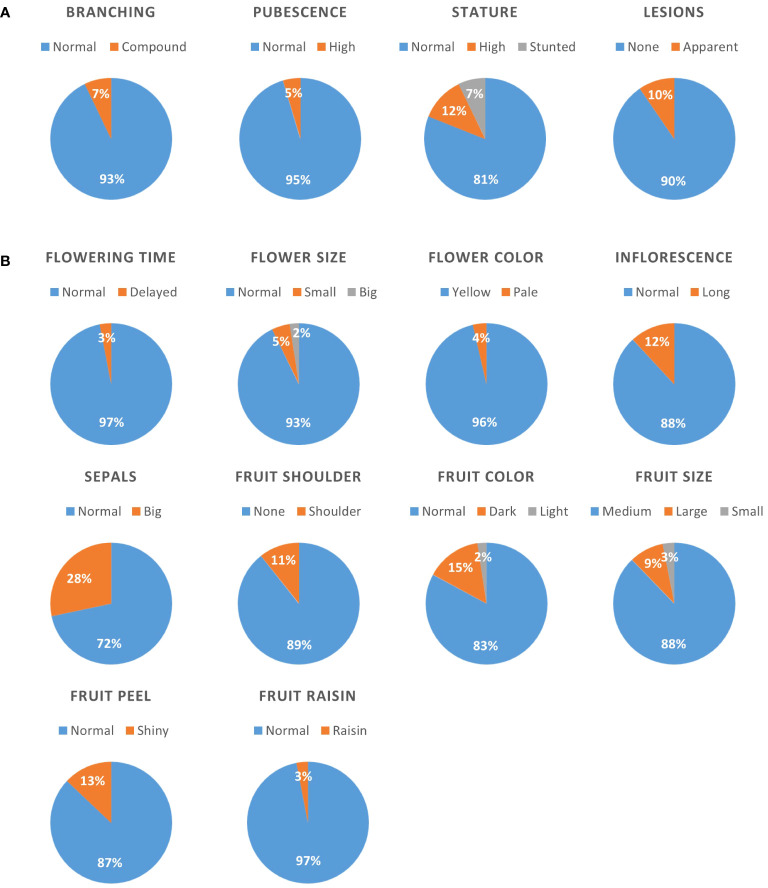
Qualitative characterization of the BILs population for developmental traits. All lines were scored for various traits, and the distribution is presented as pie charts for vegetative **(A)** and reproductive **(B)** traits. Numbers within the charts denote the percentage of lines presenting the specific phenotype. The phenotype of the M82 and TA209 parental lines is denoted “normal”.

All 166 lines of the BIL population and the parental lines M82 and S. *pimpinellifolium* (accession LA1589) were grown in a greenhouse. In order to evaluate developmental variation in this population, plants were qualitatively scored during the vegetative stage for the following traits: plant stature (normal, high or stunted), branching (normal or compound), stem pubescence (normal or high), and the occurrence of necrotic lesions (none or apparent) which are naturally more abundant in *S. pimpinellifolium*. During the reproductive stage of plant development, plants were scored for time to flowering (normal or delayed. None were found to flower earlier than normal), flowers color (yellow or pale) and size (normal size, smaller or bigger), length of inflorescences (normal or long) and sepals (normal or big). During fruit development and ripening, the following traits were evaluated; fruit color (normal, lighter or darker), fruit size (normal – medium, small or large), fruit shoulders at the green stage (apparent or not), appearance of the peel (shiny or not), and weather the fruits become dehydrated on the plant (normal or raisin). We found that for all these traits, the vast majority of lines (72–97%) presented a normal phenotype, i.e., similar to the parental M82 cultivar (**Figure 1**). The trait that showed the highest non-M82 rate was sepal size, with 28% of all lines presenting over-sized sepals. The most infrequent phenotypes were late flowering and raisin-like fruits, each found in 3% of all lines. In ten out of 13 traits examined, we found a non-parental phenotype. These are, stunted stature (7% of all lines), big flowers (2%), pale flower color (4%), the appearance of fruit shoulders (11%), and shiny fruit peel (13%) (**Figure 1**).

### 3.2 Heat stress-related phenotypic variation in the BILs population

**Figure 2:**
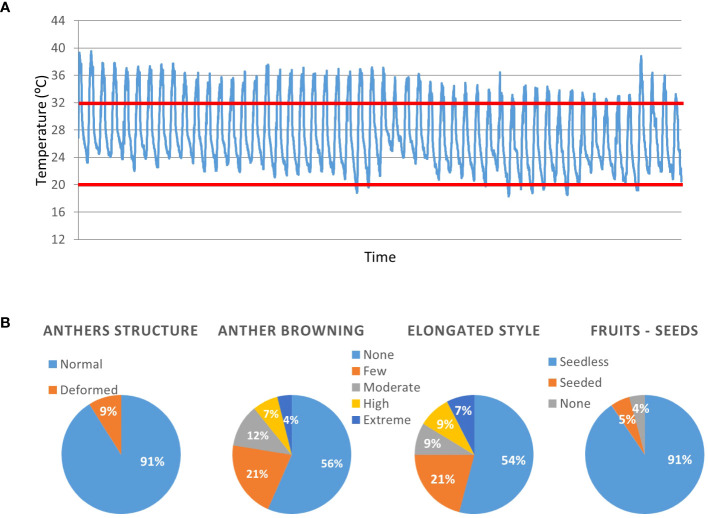
Qualitative characterization of the BILs population for heat stress related traits. All lines were grown under non-controlled, moderate chronic heat stress (MCHS) conditions **(A)** and scored for reproductive traits **(B)**. The heat-stress day and night temperature thresholds are denoted by red lines. Numbers within the charts denote the percentage of lines presenting the specific phenotype. The phenotype of the M82 and TA209 parental lines is denoted “normal”.

In order to evaluate the adequacy of the *S. pimpinellifolium* BILs population for the identification of genetic elements linked with heat tolerance traits, all lines were grown in a greenhouse during the summer. Throughout the reproductive phase of the plants, day temperatures did not go below 32°C, and night temperatures were usually above 20°C, creating conditions of moderate chronic heat stress (MCHS, **Figure 2A**). Under these conditions, we monitored inflorescences for the occurrence of heat stress related phenotype, i.e., anther deformation, anther browning, style elongation and seeds production (**Figure 2B**; **Supplementary Figure S1**). We found that the anther structural deformations appeared in 9% of the lines. For anthers browning and style elongation we used a 5-levels scale, from “none” to “extreme” according to the severity of the phenotype. In approximately half of the lines (56% and 54% for anther browning and style elongation, respectively), flowers were normal, with no stress-related impairments. However, 11% of the lines presented high or extreme occurrence of anther browning, and style elongation was intensely observed in 16% of the lines. The most drastic effect of the high temperatures was observed in seed production. Almost all lines (91%) produced seedless fruits. Few lines (4%) did not produce fruits at all, and only 5% of all lines were able to produce seeds under these growth conditions (**Figure 2**; **Supplementary Figure S1**).

**Figure 3:**
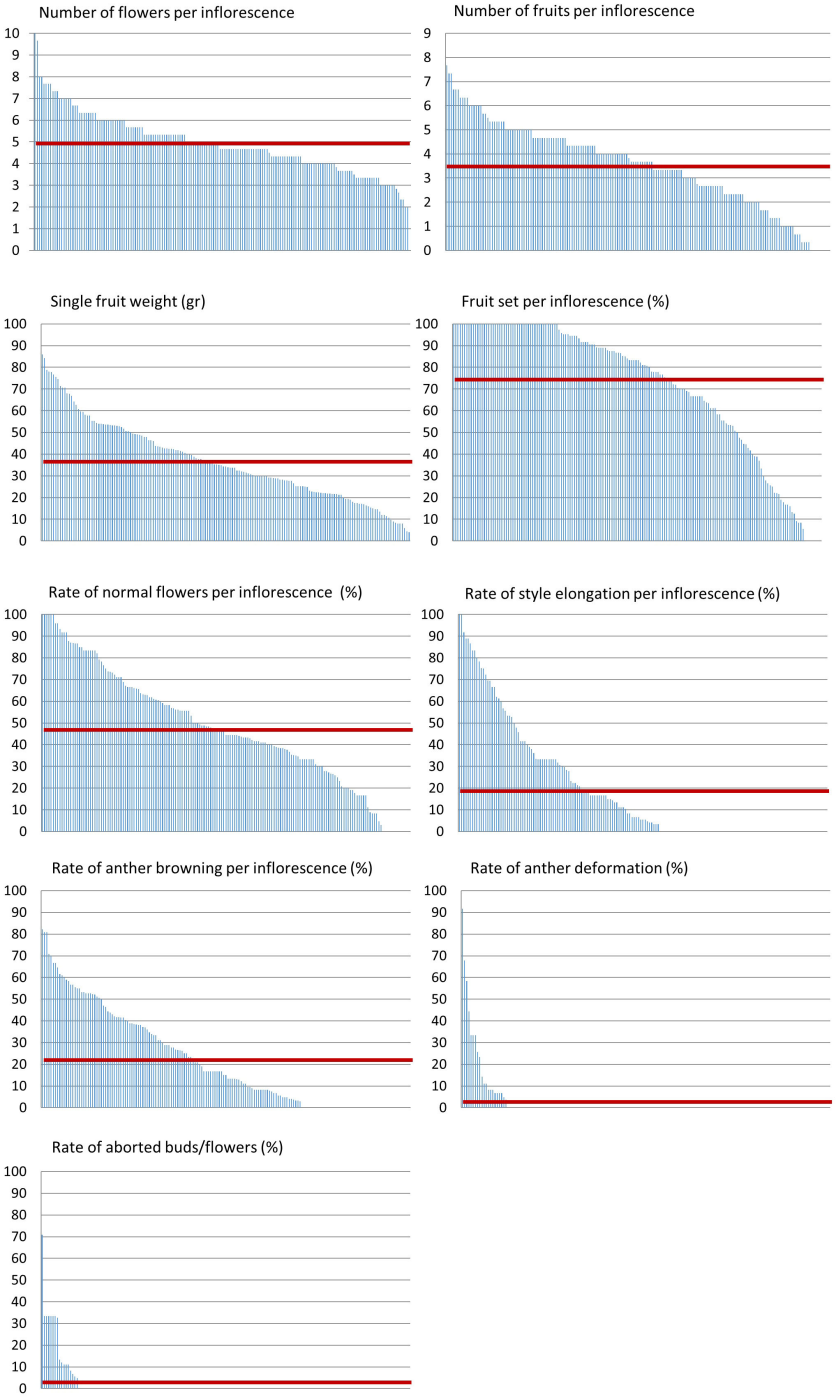
Phenotypic distribution of developmental and stress-related traits. All lines (X axis) were grown under non-controlled heat stress conditions and scored for developmental and heat stress related traits depicted in the charts’ titles. The average value per trait is marked by a red line.

Additionally, we measured several quantitative traits and examined their distribution across the lines. Among these are the number of flowers per inflorescence, number of fruits per inflorescence, and single fruit weight (**Figure 3**). Traits known to be related to heat stress response include the rate of normal flowers, flowers with elongated style, flowers with browning of anthers, flowers with anther deformations, aborted buds or flowers, and the rate of fruit set - all scored per inflorescence (**Figure 3**). We found a wide distribution for all traits examined. However, only a small proportion of the population presented heat stress sensitivity in terms of anther deformation and aborted buds/flowers. On the contrary, fruit set rate, which is an important yield trait strongly affected by heat stress, presented a very wide distribution (**Figure 3**; **Table 1**). Out of the 160 lines scored for this trait, we found all levels of fruit set, from zero to 100%, with an average of 74.8% (**Table 1**). Considering the M82 value, which is 68.6% under these conditions, our results suggest that there are several lines with improved fruit set under heat stress conditions.

**Table 1:** Phenotypic characterization of the BILs population - quantitative traits statistics.

Trait	MCHS - related	Min	Max	Avg	Med	CV (%)	M82	SP
Number of flowers	No	0	10	4.9	4.7	2.2	5.3	8.0
Normal flowers (%)	Yes	0	100	47.8	46.1	4.3	86.7	74.4
Anther browning (%)	Yes	0	82.2	21.7	15.0	7.9	0	0
Elongated style (%)	Yes	0	100	19.4	5.6	10.5	13.3	47.8*
Anther deformation (%)	Yes	0	91.7	3.0	0	29.4	0	0
Aborted buds (%)	Yes	0	70.8	2.3	0	29.2	0	11.1
Fruit number	No	0	7.7	3.5	3.7	3.9	3.7	0**
Fruit set (%)	Yes	0	100	74.8	83.7	2.9	68.6	0**
Single fruit weight (gr)	No	4	85.9	36.8	34.3	3.9	30.4	na

All traits were scored per inflorescence, except single fruit weight. M82 – the cultivated parent, SP – the *S. pimpinellifolium* parent (LA1589), MCHS – moderate chronic heat stress.

*elongated style is a naturally occurring phenomenon in wild species, denoting self-incompatibility. **under normal greenhouse watering, reproduction of wild species is hindered.

### 3.3 Individual lines present improved heat stress tolerance attributes

**Figure 4:**
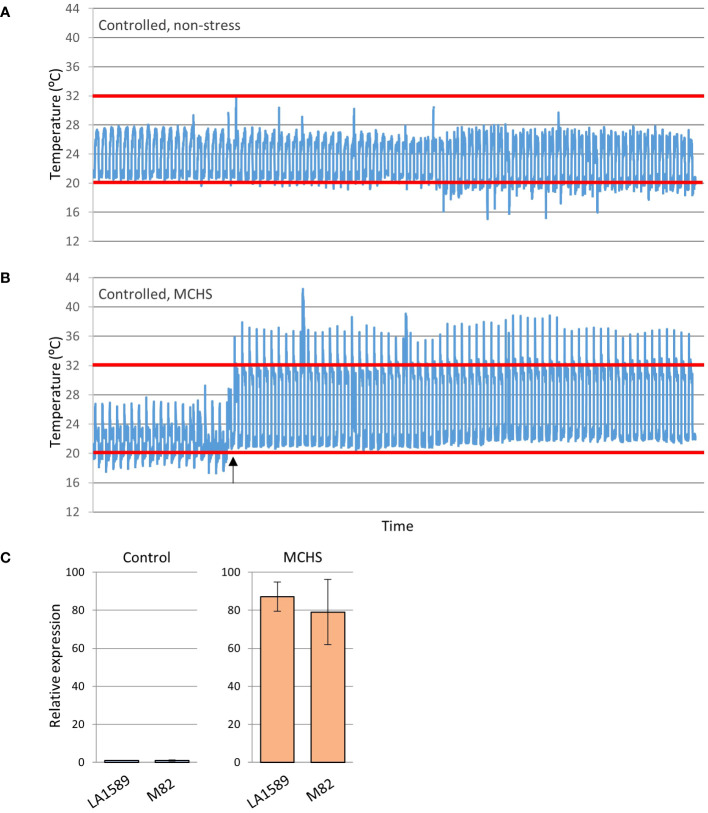
Conditions of the controlled heat stress experiment. Selected lines were grown under controlled non-stress **(A)** and moderate chronic heat stress (MCHS) **(B)** conditions. Expression of the heat-stress responsive gene Hsp17.6 was tested to validate the occurrence of heat stress in the two parental lines, M82 and LA1589 (*S. pimpinellifolium*) **(C)**. The heat-stress day and night temperature thresholds are denoted by red lines. Black arrow marks the initiation of heat stress.

**Figure 5:**
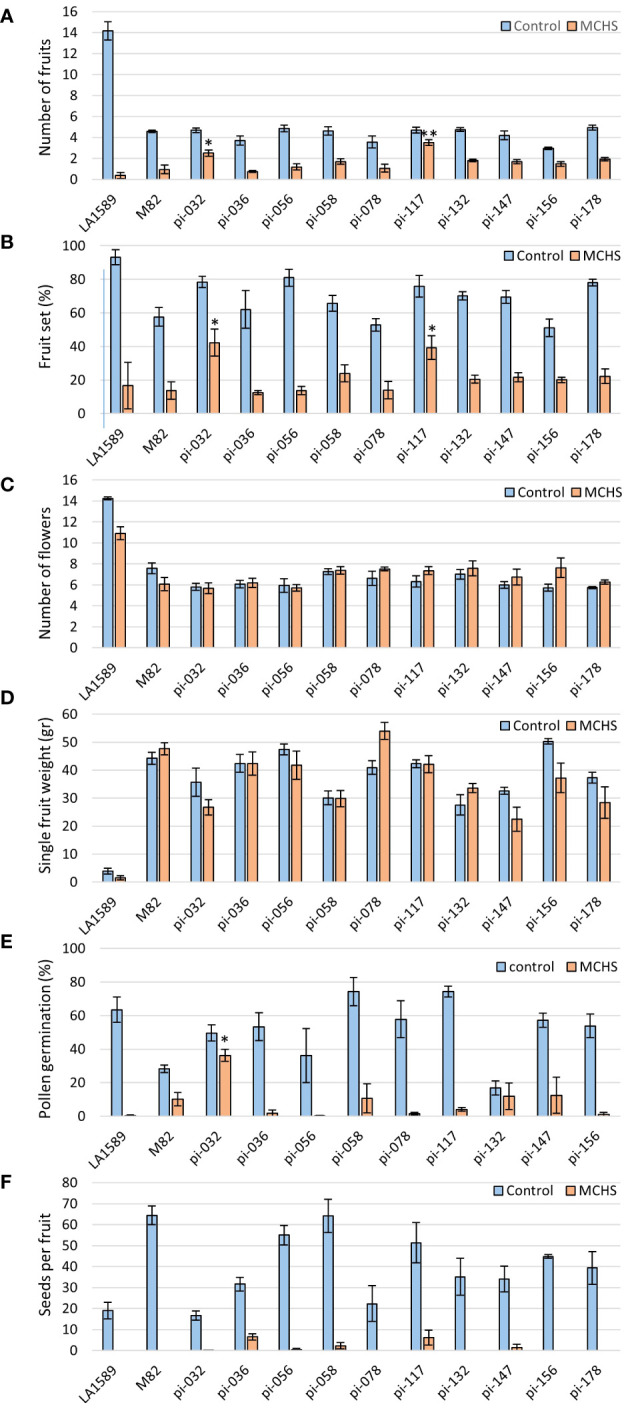
The effect of moderate chronic heat stress (MCHS) on the number of fruits **(A)**, fruit-set rate **(B)**, number of flowers **(C)**, single fruit weight **(D)**, pollen germination **(E)**, and number of seeds per fruit **(F)**. Selected lines were grown in parallel under control (26°C/20°C day/night) and MCHS (32°C/22°C day/night) conditions, and scored for reproductive performance. Each bar represents an average value of four plants per line. Statistical significance was tested using TTEST for each line compared with the heat stress sensitive M82 in MCHS conditions. (*) - p-value < 0.05, (**) - p-value < 0.01.

The phenotypic screen that was performed on the entire collection of *S. pimpinellifolium* BILs under heat stress conditions revealed that indeed, phenotypic variation for heat stress related traits exists in this population. In order to directly link these phenotypes to elevated temperatures and to identify specific lines with heat stress tolerance traits, we conducted an environmentally controlled experiment in which selected lines were grown in parallel under MCHS (32°C/22°C day/night) and control (26°C/20°C day/night) conditions (**Figures 4A, B**). The lines were selected based on their genetic maps to maximize genome coverage of LA1589, considering space limitation in the controlled setup (**Supplementary Figure S2**). The stress conditions were initiated at the beginning of flowering and the response of plants to the stress was confirmed on the molecular level, by validating the increased transcription of the heat stress responsive gene *Hsp17.6* exclusively in plants under MCHS conditions. This gene was almost undetectable in plants grown under control, non-stress conditions (**Figure 4C**). When plants reached full flowering and fruit development stages, the effect of MCHS was highly apparent. The number of fruits per inflorescence and the rate of fruit set were dramatically reduced in MCHS relative to control conditions, for all lines tested (**Figures 5A, B**). However, when comparing the lines under MCHS conditions only, two lines were able to produce a higher number of fruits per inflorescence. Similarly, two lines (one in common) presented a significantly higher level of fruit set (**Figure 5B**). The number of flowers and single fruit weight are not always affected by heat stress and indeed, we did not detect a significant reduction in MCHS compared with control for any of the lines (**Figures 5C, D**). In addition to the traits measured in the whole-population screen experiment (**Figure 3**), in the controlled experiment we included measurements of pollen germination, and number of seeds per fruit, which are well known to be strongly affected by heat stress. Our results show an intense reduction in these traits for all lines under MCHS conditions, compared to control (**Figures 5E, F**). Pollen germination (the fraction of germinated pollen) was highly variable already under control conditions, differing between the parental lines (63.5% and 28.2% in *S. pimpinellifolium* and M82, respectively), and ranging between lines from 16.8% to 74.3%. Nonetheless, under MCHS conditions, all lines presented a sharp decrease in pollen germination, from a complete lack of germination (0%) and up to an exceptional line presenting 36.2% of pollen germination (**Figure 5E**). The most variable trait under control conditions was the number of seeds per fruit, which is linked with fruit size, where high variation is expected considering the big difference in fruit size between the parental lines. Whereas fruits from *S. pimpinellifolium* and M82 produced on average 19 and 64 seeds per fruit, respectively, under control conditions, in the BILs seeds production ranged between 17 and 64 seeds per fruit on average. Under MCHS conditions, both parental lines did not produce seeds at all, whereas two lines were able to produce a small amount of seeds (6.2 and 6.5 per fruit on average, **Figure 5F**), although the increase was not significant. Overall, we observed a significant reduction in all lines in MCHS compared with control, for all heat stress related traits (i.e. number of fruits, fruit set, pollen germination and seeds per fruit). For some traits, we could detect one or two lines that performed better than the sensitive M82 parental cultivar under MCHS conditions, further reinforcing our claim that this BIL population is suitable for mining for heat stress tolerance related lines and the future identification of heat stress QTL, given further research.

### 3.4 Three heat stress tolerant lines share a common genetic introgression

**Figure 6:**
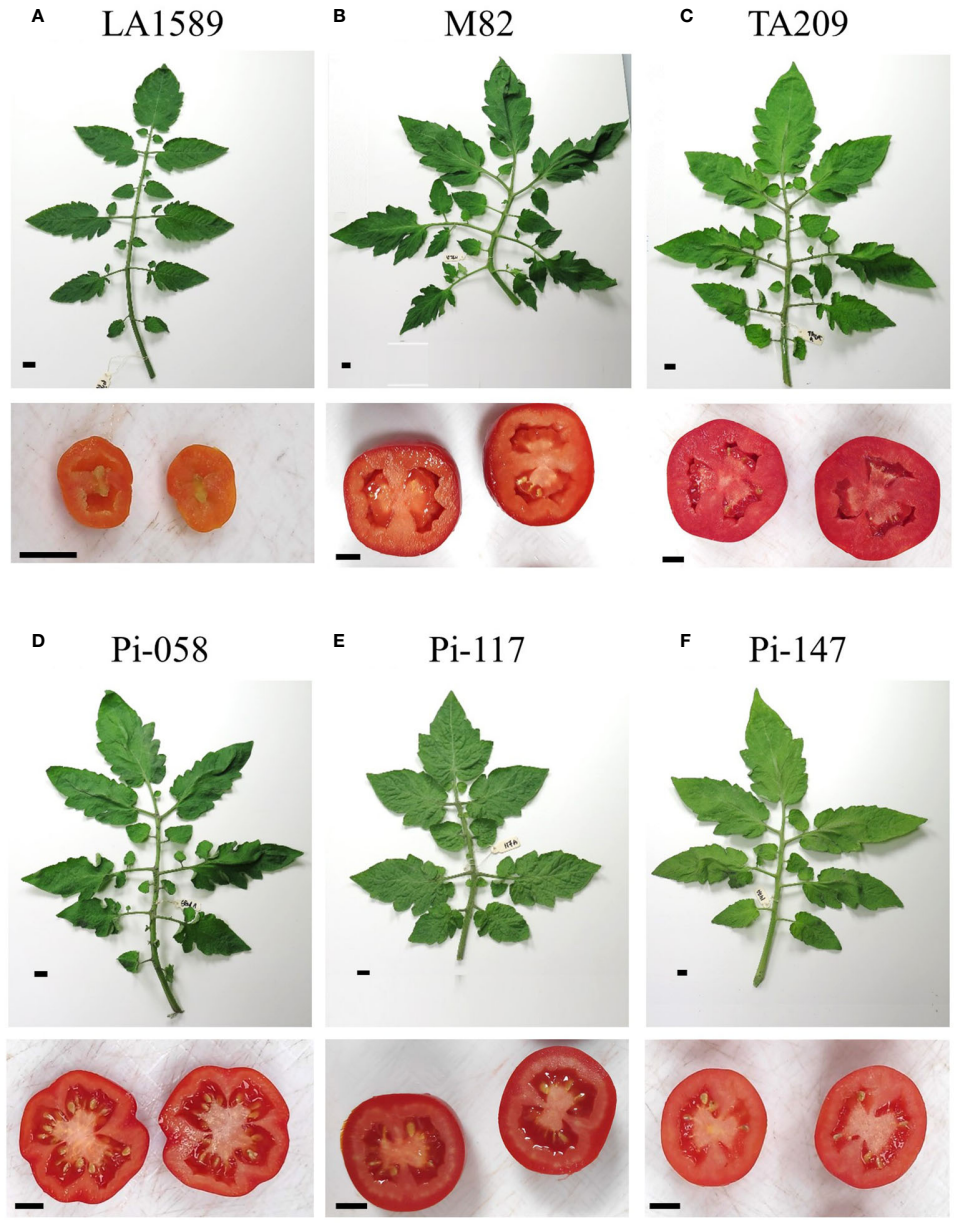
Leaf and seeds set of parental lines LA1589 **(A)**, M82 **(B)**, TA209 **(C)** and tolerant BILs pi-058 **(D)**, pi-117 **(E)** and pi-147 **(F)** under MCHS conditions. The black bar indicates 1 cm.

**Figure 7:**
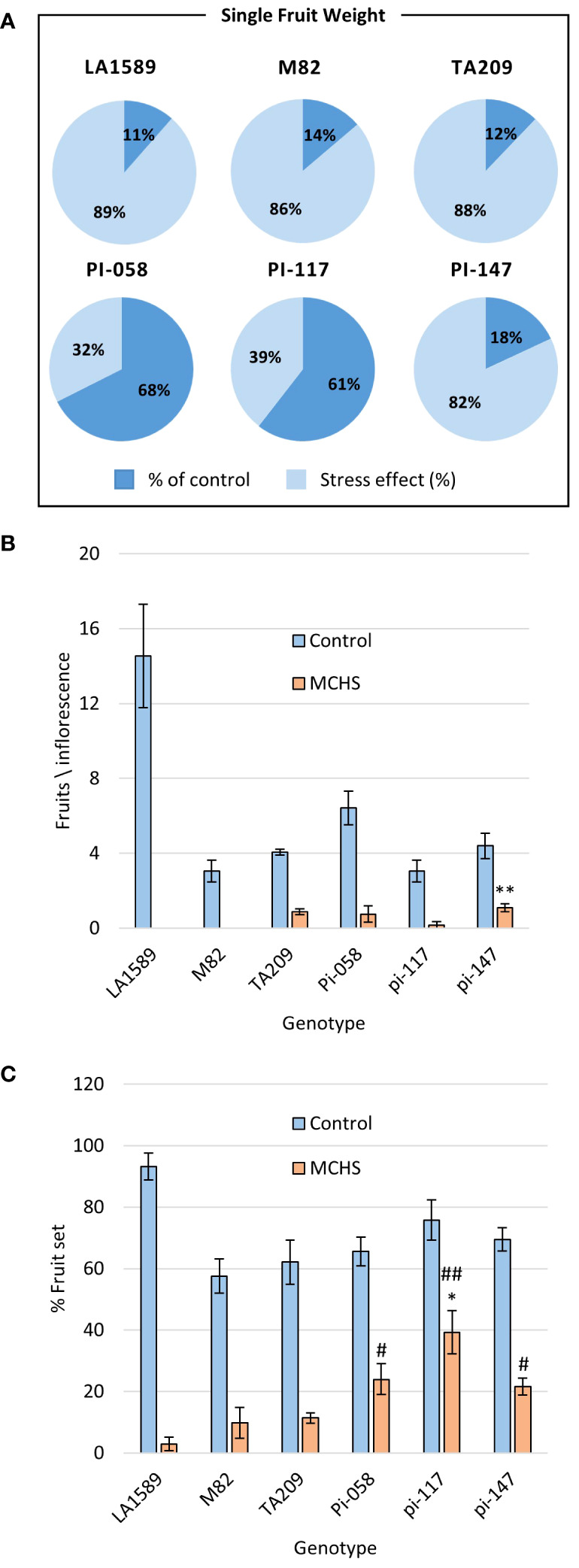
The lines pi-058, pi-117, and pi-147 present heat stress tolerance in fruit-related phenotypes. The effect of MCHS was tested for single fruit weight as a ratio of fruit weight in MCHS relative to fruit weight in control conditions **(A)**, number of fruits per inflorescence **(B)**, and fruit-set rate **(C)** Using Student’s TTEST compared with M82 and TA209 separately. (*), p-value < 0.05 compared to M82. (**), p-value < 0.01 compared to M82. (#), p-value < 0.05 compared to TA209. (##), p-value < 0.01 compared to TA209.

**Figure 8:**
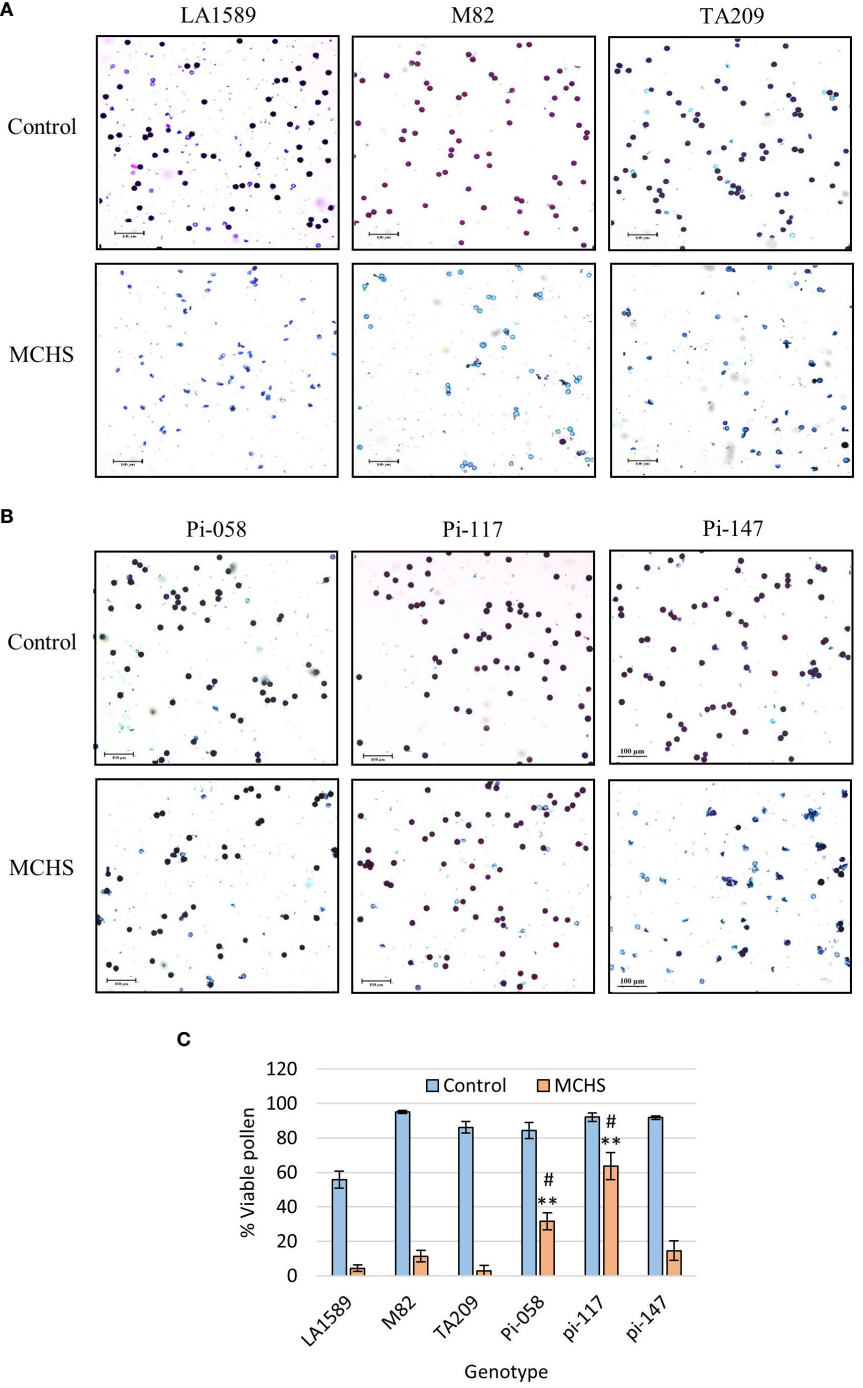
Pollen viability in lines pi-058, pi-117, and pi-147. Differential Alexander staining was performed on pollen from flowers sampled from plants grown under control or MCHS conditions. Viable pollen is round and stained purple, while unviable pollen is light blue and frequently mis-shaped. The frequency of viable pollen is reduced in MCHS conditions in all lines, as shown in representative figures presenting the LA1589, M82 and TA209 parental lines **(A)**, the pi-058, pi-117, pi-147 lines **(B)**, and quantification of the results for all lines **(C)**. (**), p-value < 0.01 compared to M82. (#), p-value < 0.05 compared to TA209.

In the course of the controlled heat stress experiment, we identified several lines that were performing better than parental lines under MCHS conditions, for several traits. These lines, named pi-058, pi-117 and pi-147, presented normal vegetative development and leaf shape (**Figure 6**), but were among the only lines that were able to produce seeds under MCHS conditions (**Figure 6**). In order to validate this observation, we carried out controlled heat stress experiments including the TA209 line that was used for a backcross step during the construction of the BILs population, hence is considered another cultivated parental line, in addition to M82. Using the same set-up and conditions, MCHS was initiated at flowering (**Figure 4**) and we scored the plants for number of fruits per inflorescence, fruit weight, fruit-set, and pollen viability. We found that fruit weight of pi-058 and pi-117 was much less affected by the stress compared with parental lines – presenting an average reduction of 32% and 39%, respectively, while M82 reached 86% reduction in fruit weight and TA209 88% reduction. LA1589 did not produce fruits in these conditions (**Figure 7A**). Pi-147 was more similar to the M82 and TA209 parental lines in terms of single fruit weight, however, it did perform significantly better in terms of number of fruits per inflorescence, while pi-058 and pi-117 did not produce more fruits compared to either M82 or TA209 (**Figure 7B**). Importantly, all three lines, i.e. pi-058, pi-117 and pi-147 reached higher fruit-set rates under MCHS conditions. While the parental lines did not go beyond 11.38% (TA209), fruit set rates in pi-058, pi-117 and pi-147 were 23.95%, 39.29% and 21.6%, respectively (**Figure 7C**), confirming their heat-stress tolerance. The levels of pollen viability were as well adversely affected by the stress treatment, being reduced to 3–11.3% in the parental lines. However, pi-117 maintained a very high rate of viable pollen (63.75%) under these conditions, as well as pi-058 that reached 31.66%. The line pi-147 reached 14.67%, which was not significantly higher than either M82 or TA209 (**Figure 8**).

**Figure 9:**
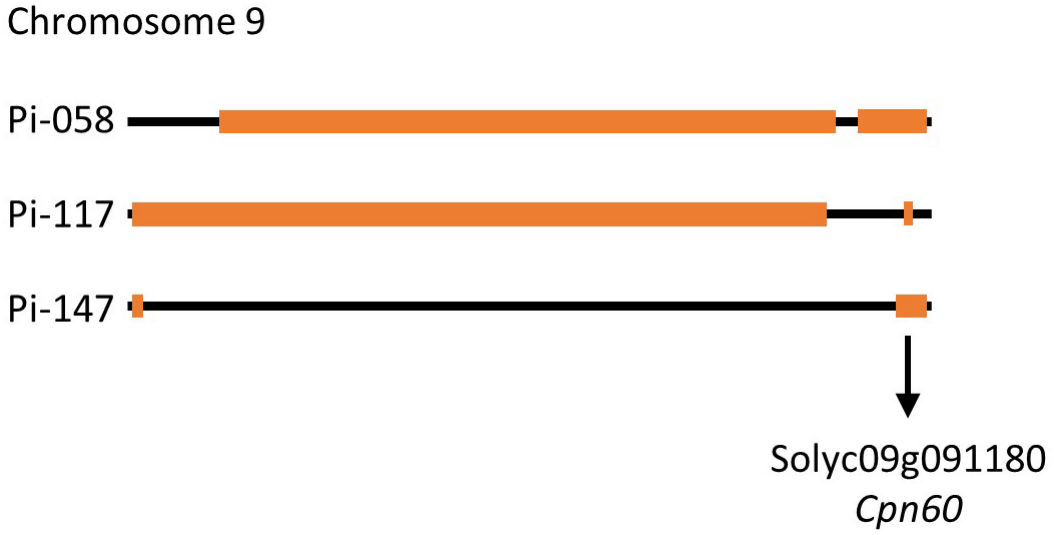
Schematic representation of chromosome 9 in lines pi-058, pi-117 and pi-147. Orange boxes mark the LA1589 *S. pimpinellifolium* introgressions. The black arrow indicated the common introgression containing the Cpn60 chaperonin gene.

Although heat stress tolerance is a highly complex trait, it is controlled by a finite number of regulators, which activate the heat stress response. Therefore, it is possible that common factors are controlling the tolerance effect of pi-058, pi-117 and pi-147, mainly observed by the high fruit-set rate under MCHS conditions (**Figure 7C**). Utilizing the genetic maps of these lines, we identified a single common introgression of the LA1589 genome at the end of chromosome 9, located between 65.5 and 66.3Mbp (**Figure 9**; **Table 2**). This 0.8Mbp region contains 128 annotated genes (**Supplementary Table 1**). GO term enrichment analysis (https://amigo.geneontology.org/) revealed that GO:0009408 - response to heat – was highly enriched in this group of genes (p-value = 1.37E-06, corrected p-value = 1.41E-04), including the *Cpn60* Chaperonin (*Solyc09g091180*, **Table 3**), which is known to contribute to heat stress tolerance.

**Table 2:** Physical location of LA1589 introgressions in chromosome 9 of the pi-058, pi-117 and pi-147 lines.

Line	Introgression no.	Length Mbp, (SL2.4)	Start End Mbp, (SL2.4)
pi-058	1	52.20	7.80
			60.00
	2	5.90	61.80
			67.70
pi-117	1	58.65	0.35
			59.00
	**2**	**0.80**	**65.50**
			**66.30**
pi-147	1	0.85	0.05
			0.90
	2	2.60	65.10
			67.70

The second introgression in pi-117 (Bold) is common between all three lines.

**Table 3:** Genes with the GO term ‘response to heat’ that are found in the common 0.8Mbp introgression.

Locus name	Description
Solyc09g090700	Aldehyde dehydrogenase
Solyc09g090870	DNA mismatch repair
Solyc09g091180	Chaperonin (Cpn60)
Solyc09g090890	DNA mismatch repair protein
Solyc09g091660	ABC transporter
Solyc09g091670	ATP-binding cassette transporter
Solyc09g091830	Peptidoglycan binding domain containing protein

Overall, our results demonstrate the suitability of the presented BIL population derived from *S. pimpinellifolium* wild-species accession LA1589 and the tomato cultivars M82 and TA209 for heat stress related research and possible future identification of QTL for heat tolerance in tomato as a mean to mitigate the negative impact of increased temperatures on tomato development and productivity.

## 4 Discussion

The increased ambient temperatures in many agricultural regions around the world have detrimental influence on crop yield, therefore the development of heat stress resistant cultivars is of upmost importance. Utilizing the broad genetic variation that exists in wild species is a prevalent approach for the identification of genes and alleles that improve crops’ performance under harsh environmental conditions, including heat stress (Zamir, 2001). In order to evaluate a new BILs population generated from a cross between M82 (*S. lycopersicum*) and the *S. pimpinellifolium* LA1589 accession for its relevance as a source for the identification of heat stress tolerance genes, we screened the population under non-controlled heat stress and under controlled, moderate chronic heat stress (MCHS) conditions. Since thermotolerance is a highly complex trait, we examined several vegetative and reproductive parameters that include plant stature and flowering time, inflorescence length, flower size and color, anther structure and apparent damage, fruit color, size and seeds content. Additionally, we analyzed the number of flowers and fruits, the rate of fruit set, single fruit weight, pollen germination rate, and the number of seeds per fruit. We observed a wide range of responses for all traits tested (**Figure 3**).

Owing to the controlled experimental set up, which allowed the comparison between MCHS and control conditions, we could identify the traits that are directly affected by MCHS. These include number of fruits and fruit set rate, pollen germination rate, and the number of seeds per fruit, all are well known heat stress related traits (Peet et al., 1998; Pressman et al., 2007), thus supporting our hypothesis that this BIL population is a relevant germplasm for exploring heat stress response in tomato.

In the controlled experiments, the number of flowers and single fruit weight were generally not affected by the MCHS conditions employed. Several studies reported a reduction in flower production under high temperature conditions, but under moderate heat stress conditions, flower number seemed to be unaffected (Abdul-Baki, 1991; Xu et al., 2017). Others show that there is no significant decrease in the number of flowers under heat treatment compared to the control (Peet et al., 1997; Sato et al., 2006; Gonzalo et al., 2020). Fruit weight was also reported to be reduced up to 83% under heat stress conditions (Peet et al., 1998). These differences may be attributed to the heat stress conditions employed, i.e., acute stress versus moderate chronic stress. Moreover, the response to the different stress regimes is genotype-dependent. Style protrusion is highly variable between lines and cultivars as the frequency of this trait may range from 12.5% to 80% (Levy et al., 1978). Notably, this phenomenon is naturally occurring in wild relatives of tomato (Chen et al., 2007) and therefore cannot be considered as a pure heat stress related trait in the *S. pimpinellifolium* population. Nevertheless, our phenotypic screen revealed that about half of the lines (54%) did not present style protrusion at all, and 25% of the lines presented moderate (9%), high (9%), or extreme (7%) level of style protrusion. Levy et al. (1978) reported up to 66% flower buds drop rate in a tomato heat sensitive cultivar. Our population screen results show that although most lines did not present buds or flowers abortion, 16 BILs presented varying abortion rates, ranging between 5% and 70%. Since pollen grain is the most thermosensitive organ (Zinn et al., 2010), special attention is given to pollen quality and performance under heat stress conditions. Pressman et al. (2002) reported a reduction of 62% and 92%, in pollen viability and germination, respectively. Similarly, Firon et al. (2006) showed a 29–78% decrease in pollen viability and 80–90% decrease in pollen germination as a result of heat stress. Our results indicate as well a very strong reduction in pollen viability and germination under MCHS conditions. While some BILs completely lost germination capability, the best performing lines presented a decrease of 32% in pollen germination compared with reductions of 80% in the M82 parental line.

It was very clear throughout our experiments, that LA1589, the parental *S. pimpinellifolium* wild-species itself, was not more tolerant than the cultivated tomato lines, in any of the traits tested. In fact, it was the most sensitive. This can partially explained by the unnatural environment in the greenhouse which includes detached soil growth media, and consistent watering and fertilization for homogenous treatment to all plants across all experiments, but are actually stressful for the wild-adapted *S. pimpinellifolium*. Nonetheless, the hypersensitivity of LA1589 to heat stress in our hands, together with the wide variation in heat-stress related traits among the BILs, and the identification of three tolerant lines, suggest that epistatic effects contribute to the observed heat stress tolerance.

Overall, our results highlight the relevance and importance of this BIL population as a new resource for the identification of heat stress related tolerance genes and QTLs. This population was never tested for heat stress related traits. Specifically, the genetic structure of this population may facilitate the identification of candidate genes or QTLs by aligning genetic maps of phenotypically similar lines, as demonstrated by the phenotypic and genetic comparison of pi-058, pi-117 and pi-147 (**Figures 7**–**9**; **Supplementary Figure S3**). In the analysis presented here, we identified a 0.8 Mbp region on chromosome 9 found exclusively in lines pi-058, pi-117 and pi-147 and absent in the other, non-tolerant lines (**Supplementary Figure S3**), which was enriched for genes related to heat stress response, thus these may be involved in the observed heat stress tolerance of pi-058, pi-117 and pi-147. This introgression includes seven genes related to heat stress response (**Table 3**), some of them are known to contribute to tolerance under heat stress conditions. For example, the chaperonin 60 (*Cpn60*, *Solyc09g091180*) was found to be overexpressed in thermotolerant tomato that presents high pollen viability under heat stress (Mazzeo et al., 2018) and to support photosystem II under conditions of heat stress (Preczewski et al., 2000). Two additional genes found in the common introgression, *Solyc09g091660* and *Solyc09g091670*, encode ABC transporters responsible for heat stress tolerance by maintaining cell membrane integrity under heat stress conditions. These genes also regulate the transport, distribution and accumulation of ABA and secondary metabolites in different organs of the plant, which in turn modulate thermotolerance (Dahuja et al., 2021). *Solyc09g090870* and *Solyc09g090890* are involved in DNA mismatch repair which plays an important role in DNA repair following heat stress (Kantidze et al., 2016). Therefore, although *Cpn60* is an immediate candidate for supporting the heat stress tolerance of pi-058, pi-117 and pi-147, it is possible that a joint activity of all or some of these seven genes (**Table 3**) are responsible for the observed effect. Moreover, this introgression may not be the sole effector that promotes thermotolerance, as epistatic effects may also take place, as mentioned here before.

It is interesting to note that among the three thermotolerant lines identified in this study, pi-147 is, for some traits, not performing as well as pi-058 and pi-117 which are highly similar (**Figures 7A**, **8**). Since pi-058 and pi-117 contain another common introgression on chromosome 9 which is absent in pi-147 (**Figure 9**), genes found in this region may be driving this difference, an hypothesis that requires further studies. Since in our experimental setup, LA1589 was not performing well under MCHS conditions, having evolved in harsh conditions, it is unlikely that the heat stress tolerance of specific BILs will be attributed to a single introgression. It is possible though, that a specific interaction between a single introgression and the cultivated genome will generate a causal network for tolerance. This should be further studied considering the common introgression on chromosome 9 (**Figure 9**).

Taking into consideration other publications demonstrating a beneficial contribution of *S. pimpinellifolium* accessions to heat stress tolerance, we suggest that this BIL population may be useful for the identification of heat stress related QTLs. Introgression Lines (IL) population originated from a cross between a cultivated tomato and the *S. pimpinellifolium* TO-937 accession was used in a study that successfully identified 22 QTLs involved in reproductive traits under high temperatures. For example, fruit-set was reduced by less than 50% in several RILs while in the Moneymaker background, reduction was up to 87% (Gonzalo et al., 2020). Moreover, the specific *S. pimpinellifolium* accession LA1589 was also suggested to contribute to heat stress tolerance as two clusters of QTLs involved in the responses of reproductive traits to heat stress were identified in a LA1589-derived population (Gonzalo et al., 2022). For each of the heat stress related traits that were tested, we could identify specific lines that were performing significantly better than other lines, and of both parental lines. These lines will serve as a starting genetic material in future studies aiming to identify QTLs and causal genes for each of the tolerance traits. Moreover, since the controlled MCHS experiments were performed on selected lines, not including the entire population (due to space limitations), our results suggest that additional tolerant lines may be identified in future studies that will complement the MCHS controlled screen for the entire population, in a future perspective of developing heat stress tolerant tomato cultivars.

## Data availability statement

The original contributions presented in the study are included in the article/**Supplementary Material**. Further inquiries can be directed to the corresponding author.

## Author contributions

NB: Conceptualization, Formal analysis, Investigation, Writing – original draft, Writing – review & editing. GM: Formal analysis, Investigation, Methodology, Project administration, Supervision, Writing – review & editing. TD-M: Formal analysis, Investigation, Writing – review & editing. AB: Formal analysis, Investigation, Writing – review & editing. BO: Writing – review & editing, Funding acquisition. DZ: Writing – review & editing, Resources. ML-L: Conceptualization, Data curation, Formal analysis, Funding acquisition, Investigation, Methodology, Supervision, Validation, Visualization, Writing – original draft, Writing – review & editing.

## References

[B1] Abdul-Baki A. A. (1991). Tolerance **of** tomato cultivars and selected germplasm to heat stress. J. Am. Soc. Hortic. Sci. 116, 1113–1116. doi: 10.21273/JASHS.116.6.1113

[B2] Alexander M. P. (1980). A versatile stain for pollen fungi, yeast and bacteria. Stain Technol. 55, 13–18. doi: 10.3109/10520298009067890 6158141

[B3] Ayenan M. A. T. Danquah A. Hanson P. Ampomah-Dwamena C. Sodedji F. A. K. Asante I. K.. (2019). Accelerating Breeding for Heat Tolerance in Tomato (*Solanum lycopersicum L.*): An integrated approach. Agronomy 9, 720. doi: 10.3390/agronomy9110720

[B4] Bell J. Duffy P. Covey C. Sloan L. (2000). Comparison of temperature variability in observations and sixteen climate model simulations. Geophys. Res. Lett. 27, 261–264. doi: 10.1029/1999GL006080

[B5] Bita C. E. Gerats T. (2013). Plant tolerance to high temperature in a changing environment: scientific fundamentals and production of heat stress-tolerant crops. Front. Plant Sci. 4. doi: 10.3389/fpls.2013.00273 PMC3728475 23914193

[B6] Charles W. B. Harris R. E. (1972). Tomato fruit-set at high and low temperatures. Plant Sci. 52, 497–506. doi: 10.4141/cjps72-080

[B7] Chen K.-Y. Cong B. Wing R. Vrebalov J. Tanksley S. D. (2007). Changes in regulation of a transcription factor lead to autogamy in cultivated tomatoes. Science 318, 643–645. doi: 10.1126/science.1148428 17962563

[B8] Conesa M.À. Muir C. D. Roldán E. J. Molins A. Perdomo J. A. Galmés J. (2017). Growth capacity in wild tomatoes and relatives correlates with original climate in arid and semi-arid species. Environ. Exp. Bot. 141, 181–190. doi: 10.1016/j.envexpbot.2017.04.009

[B9] Dahuja A. Kumar R. R. Sakhare A. Watts A. Singh B. Goswami S.. (2021). Role of ATP-binding cassette transporters in maintaining plant homeostasis under abiotic and biotic stresses. Physiologia Plantarum 171, 785–801. doi: 10.1111/ppl.13302 33280130

[B10] Doganlar S. Frary A. Ku H.-M. Tanksley S. D. (2002). Mapping quantitative trait loci in inbred backcross lines of *Lycopersicon pimpinellifolium* (LA1589). Genome 45, 1189–1202. doi: 10.1139/g02-091 12502266

[B11] Driedonks N. Wolters-Arts M. Huber H. de Boer G.-J. Vriezen W. Mariani C.. (2018). Exploring the natural variation for reproductive thermotolerance in wild tomato species. Euphytica 214, 1–12. doi: 10.1007/s10681-018-2150-2

[B12] Firon N. Shaked R. Peet M. M. Pharr D. M. Zamski E. Rosenfeld K.. (2006). Pollen grains of heat tolerant tomato cultivars retain higher carbohydrate concentration under heat stress conditions. Scientia Hortic. 109, 212–217. doi: 10.1016/j.scienta.2006.03.007

[B13] Foolad M. R. Zhang L. P. Lin G. Y. (2001). Identification and validation of QTLs for salt tolerance during vegetative growth in tomato by selective genotyping. Genome 44, 444–454. doi: 10.1139/g01-030 11444704

[B14] Gonzalo M. J. Li Y. C. Chen K. Y. Gil D. Montoro T. Nájera I.. (2020). Genetic control of reproductive traits in tomatoes under high temperature. Front. Plant Sci. 11. doi: 10.3389/fpls.2020.00326 PMC7193983 32391023

[B15] Gonzalo M. J. da Maia L. C. Nájera I. Baixauli C. Giuliano G. Ferrante P.. (2022). Genetic control of reproductive traits under different temperature regimes in inbred line populations derived from crosses between *s. Pimpinellifolium* and *s. Lycopersicum* accessions. Plants 2022 11, 1069. doi: 10.3390/PLANTS11081069 PMC9027731 35448797

[B16] Grandillo S. Tanksley S. D. (1996). QTL analysis of horticultural traits differentiating the cultivated tomato from the closely related species *Lycopersicon pimpinellifolium.* Theor. Appl. Genet. 92, 935–951. doi: 10.1007/BF00224033 24166620

[B17] Hartman J. B. St Clair D. A. (1999). Combining ability for beet armyworm, *Spodoptera exigua*, resistance and horticultural traits of selected *Lycopersicon pennellii*-derived inbred backcross lines of tomato. Plant Breed. 118, 523–530. doi: 10.1046/j.1439-0523.1999.00437.x

[B18] Hazra P. Samsul A. Sikder D. Peter K. V. (2007). Breeding tomato (*lycopersicon esculentum* mill.) Resistant to high temperature stress. Int. J. Plant Breed. 1, 31–40.

[B19] Kabelka E. Yang W. Francis D. M. (2004). Improved tomato fruit color within an inbred backcross line derived from *Lycopersicon esculentum* and L. hirsutum involves the interaction of loci. J. AMER. Soc Hortic. Sci. 129, 250–257. doi: 10.21273/JASHS.129.2.0250

[B20] Kantidze O. L. Velichko A. K. Luzhin A. V. Razin S. V. (2016). Heat stress-induced dna damage. Acta Naturae 8, 75. doi: 10.32607/20758251-2016-8-2-75-78 27437141 PMC4947990

[B21] Kimura S. Sinha N. (2008). Tomato (*Solanum lycopersicum*): a model fruit-bearing crop. Cold Spring Harbor Protoc. 3, 1–9. doi: 10.1101/pdb.emo105 21356708

[B22] Levy A. Rabinowitch H. D. Kedar N. (1978). Morphological and physiological characters affecting flower drop and fruit set of tomatoes at high temperatures. Euphytica 27, 211–218. doi: 10.1007/BF00039137

[B23] Lin G. Y. Foolad M. R. Zhang L. P. (2002). “QTL comparison of salt tolerance during seed germination and vegetative stage in a Lycopersicon esculentum x L. pimpinellifolium RIL population,” in XXVI International Horticultural Congress: Environmental Stress and Horticulture Crops, vol. 618, 59–67. doi: 10.17660/ActaHortic.2003.618.5

[B24] Liu B. Song L. Deng X. Lu Y. Lieberman-Lazarovich M. Shabala S.. (2023). Tomato heat tolerance: progress and prospects. Scientia Hortic. 322, 112435. doi: 10.1016/j.scienta.2023.112435

[B25] Lohar D. P. Peat W. E. (1998). Floral characteristics of heat-tolerant and heat-sensitive tomato (*Lycopersicon esculentum* Mill.) cultivars at high temperature. Scientia Hortic. 73, 53–60. doi: 10.1016/S0304-4238(97)00056-3

[B26] Mazzeo M. F. Cacace G. Iovieno P. Massarelli I. Grillo S. Siciliano R. A. (2018). Response mechanisms induced by exposure to high temperature in anthers from thermo-tolerant and thermo-sensitive tomato plants: A proteomic perspective. PloS One 13, e0201027. doi: 10.1371/journal.pone.0201027 30024987 PMC6053223

[B27] Miller J. C. Tanksley S. D. (1990). RFLP analysis of phylogenetic relationships and genetic variation in the genus Lycopersicon. Theor. Appl. Genet. 80, 437–448. doi: 10.1007/BF00226743 24221000

[B28] Müller F. Xu J. Kristensen L. Wolters-arts M. de Groot P. F. M. Jansma S. Y.. (2016). High-temperature-induced defects in tomato (*Solanum lycopersicum*) anther and pollen development are associated with reduced expression of B-Class floral patterning genes. PloS One 11, e0167614. doi: 10.1371/journal.pone.0167614 27936079 PMC5147909

[B29] Pease J. B. Haak D. C. Hahn M. W. Moyle L. C. (2016). Phylogenomics reveals three sources of adaptive variation during A rapid radiation. PloS Biol. 14, 1–24. doi: 10.1371/journal.pbio.1002379 PMC4752443 26871574

[B30] Peet M. M. Sato S. Gardner R. G. (1998). Comparing heat stress effects on male-fertile and male-sterile tomatoes. Plant Cell Environ. 21, 225–231. doi: 10.1046/j.1365-3040.1998.00281.x

[B31] Peet M. M. Willits D. H. Gardner R. (1997). Response of ovule development and post-pollen production processes in male-sterile tomatoes to chronic, sub-acute high temperature stress. J. Exp. Bot. 48, 101–111. doi: 10.1093/jxb/48.1.101

[B32] Peralta I. E. Spooner D. M. (2000). Classification of wild tomatoes: a review. Tomo 28, 45–54. Available at: http://hdl.handle.net/11336/152176.

[B33] Peralta I. E. Spooner D. M. Knapp S. (2008). Taxonomy of wild tomatoes and their relatives (Solanum sect. Lycopersicoides, sect. Juglandifolia, sect. Lycopersicon; Solanaceae). Syst. Bot. Monogr. 84.

[B34] Preczewski P. J. Heckathorn S. A. Downs C. A. Coleman J. S. (2000). Photosynthetic thermotolerance is quantitatively and positively correlated with production of specific heat-shock proteins among nine genotypes of Lycopersicon (tomato). Photosynthetica 38, 127–134. doi: 10.1023/A:1026760311255/METRICS

[B35] Pressman E. Peet M. M. Pharr D. M. (2002). The effect of heat stress on tomato pollen characteristics is associated with changes in carbohydrate concentration in the developing anthers. Ann. Bot. 90, 631–636. doi: 10.1093/aob/mcf240 12466104 PMC4240456

[B36] Pressman E. Shaked R. Firon N. (2007). Tomato response to heat stress: focus on pollen grains. Plant Stress 1, 216–227.

[B37] Rao E. S. Kadirvel P. Symonds R. C. Ebert A. W. (2013). Relationship between survival and yield related traits in Solanum pimpinellifolium under salt stress. Euphytica 190, 215–228. doi: 10.1007/s10681-012-0801-2

[B38] Renau-Morata B. Cebolla-Cornejo J. Carrillo L. Gil-Villar D. Martí R. María Jiménez-Gómez J.. (2024). Identification of *Solanum pimpinellifolium* genome regions for increased resilience to nitrogen deficiency in cultivated tomato. Scientia Hortic. 323, 112497. doi: 10.1016/j.scienta.2023.112497

[B39] Rick C. M. (1958). The role of natural hybridization in the derivation of cultivated tomatoes of western South America. Econ. Bot. 12, 346–367. doi: 10.1007/BF02860023

[B40] Rick C. M. Dempsey W. H. (1969). Position of the stigma in relation to fruit setting of the tomato. Bot. GAZ. 130, 180–186. doi: 10.1086/336488

[B41] Sato S. Kamiyama M. Iwata T. Makita N. Furukawa H. Ikeda H. (2006). Moderate increase of mean daily temperature adversely affects fruit set of *Lycopersicon esculentum* by disrupting specific physiological processes in male reproductive development. Ann. Bot. 97, 731–738. doi: 10.1093/aob/mcl037 16497700 PMC2803419

[B42] Sato S. Peet M. M. Gardner R. G. (2001). Formation of parthenocarpic fruit, undeveloped flowers and aborted flowers in tomato under moderately elevated temperatures. Scientia Hortic. 90, 243–254. doi: 10.1016/S0304-4238(00)00262-4

[B43] Sato S. Peet M. M. Thomas J. F. (2000). Physiological factors limit fruit set of tomato (Lycopersicon esculentum Mill.) under chronic, mild heat stress. Plant Cell Environ. 23, 719–726. doi: 10.1046/j.1365-3040.2000.00589.x

[B44] Sato S. Peet M. M. Thomas J. F. (2002). Determining critical pre- and post-anthesis periods and physiological processes in *Lycopersicon esculentum* Mill. exposed to moderately elevated temperatures. J. Exp. Bot. 53, 1187–1195. doi: 10.1093/jexbot/53.371.1187 11971929

[B45] Schneider C. Rasband W. Eliceiri K. (2012). NIH Image to ImageJ: 25 years of image analysis. Nat. Methods 9 (7), 671–675. doi: 10.1038/nmeth.2089 22930834 PMC5554542

[B46] Villalta I. Reina-Sanchez A. Bolarin M. C. Cuartero J. Belver A. Venema K.. (2008). Genetic analysis of Na+ and K+ concentrations in leaf and stem as physiological components of salt tolerance in Tomato. Theor. Appl. Genet. 116, 869–880. doi: 10.1007/s00122-008-0720-8 18251001

[B47] Xu J. Wolters-Arts M. Mariani C. Huber H. Rieu I. (2017). Heat stress affects vegetative and reproductive performance and trait correlations in tomato (*Solanum lycopersicum*). Euphytica 213, 156. doi: 10.1007/s10681-017-1949-6

[B48] Zamir D. (2001). Improving plant breeding with exotic genetic libraries. Nat. Rev. Genet. 2, 983–989. doi: 10.1038/35103590 11733751

[B49] Zinn K. E. Tunc-Ozdemir M. Harper J. F. (2010). Temperature stress and plant sexual reproduction: uncovering the weakest links. J. Exp. Bot. 61, 1959–1968. doi: 10.1093/jxb/erq053 20351019 PMC2917059

