# Supplementary material for: New population of Solanum pimpinellifolium backcross inbred lines as a resource for heat stress tolerance in tomato
Source: Front Plant Sci. 2024 Jul 1;15:1386824. doi: 10.3389/fpls.2024.1386824 (PMC11246914; doi:10.3389/fpls.2024.1386824)
Supplement: Supplementary file 1 [file Presentation_1.pptx]

## Slide 1
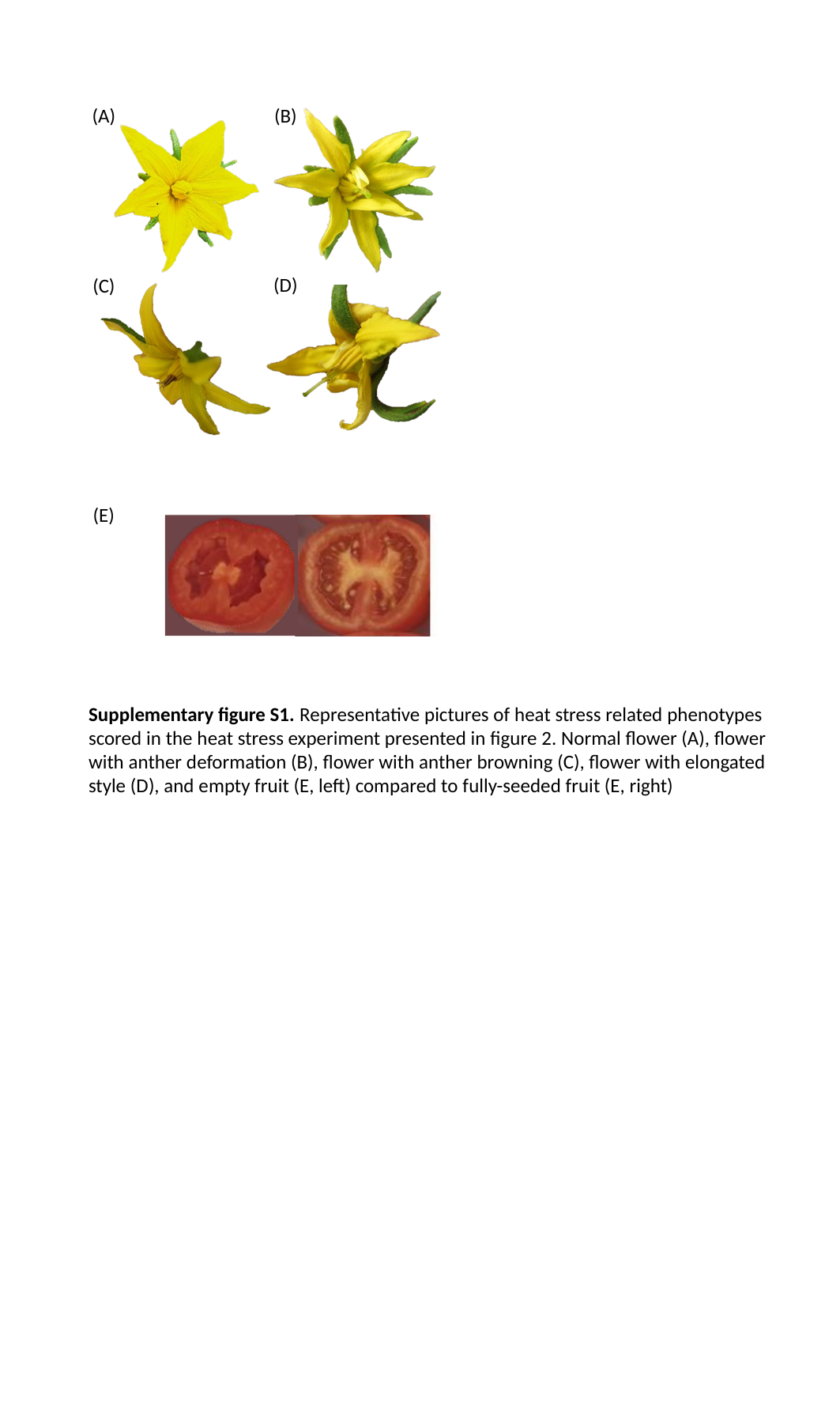

(B)
(A)
(D)
(C)
(E)
Supplementary figure S1. Representative pictures of heat stress related phenotypes scored in the heat stress experiment presented in figure 2. Normal flower (A), flower with anther deformation (B), flower with anther browning (C), flower with elongated style (D), and empty fruit (E, left) compared to fully-seeded fruit (E, right)

## Slide 2
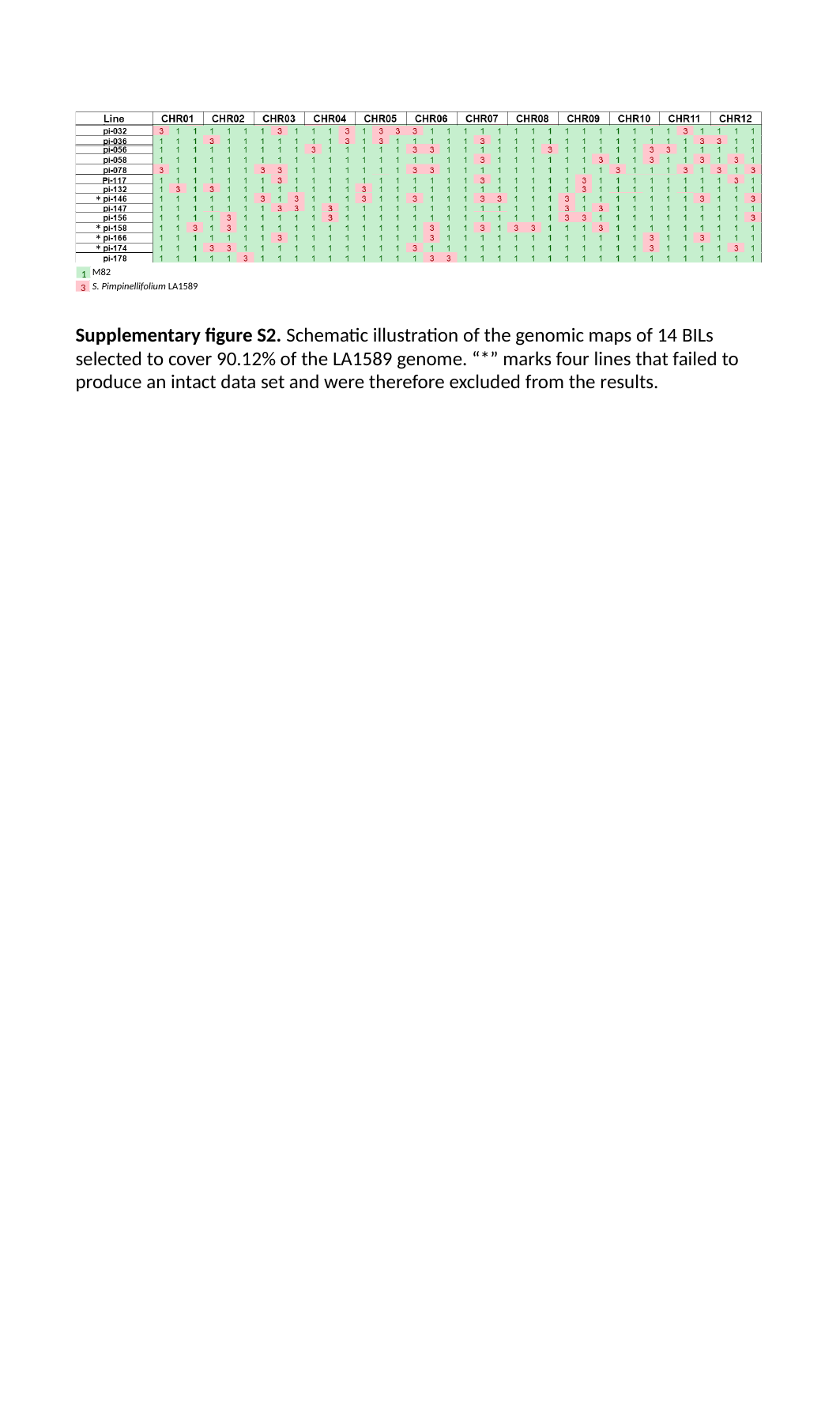

*
*
*
*
M82
1
S. Pimpinellifolium LA1589
3
Supplementary figure S2. Schematic illustration of the genomic maps of 14 BILs selected to cover 90.12% of the LA1589 genome. “*” marks four lines that failed to produce an intact data set and were therefore excluded from the results.

## Slide 3
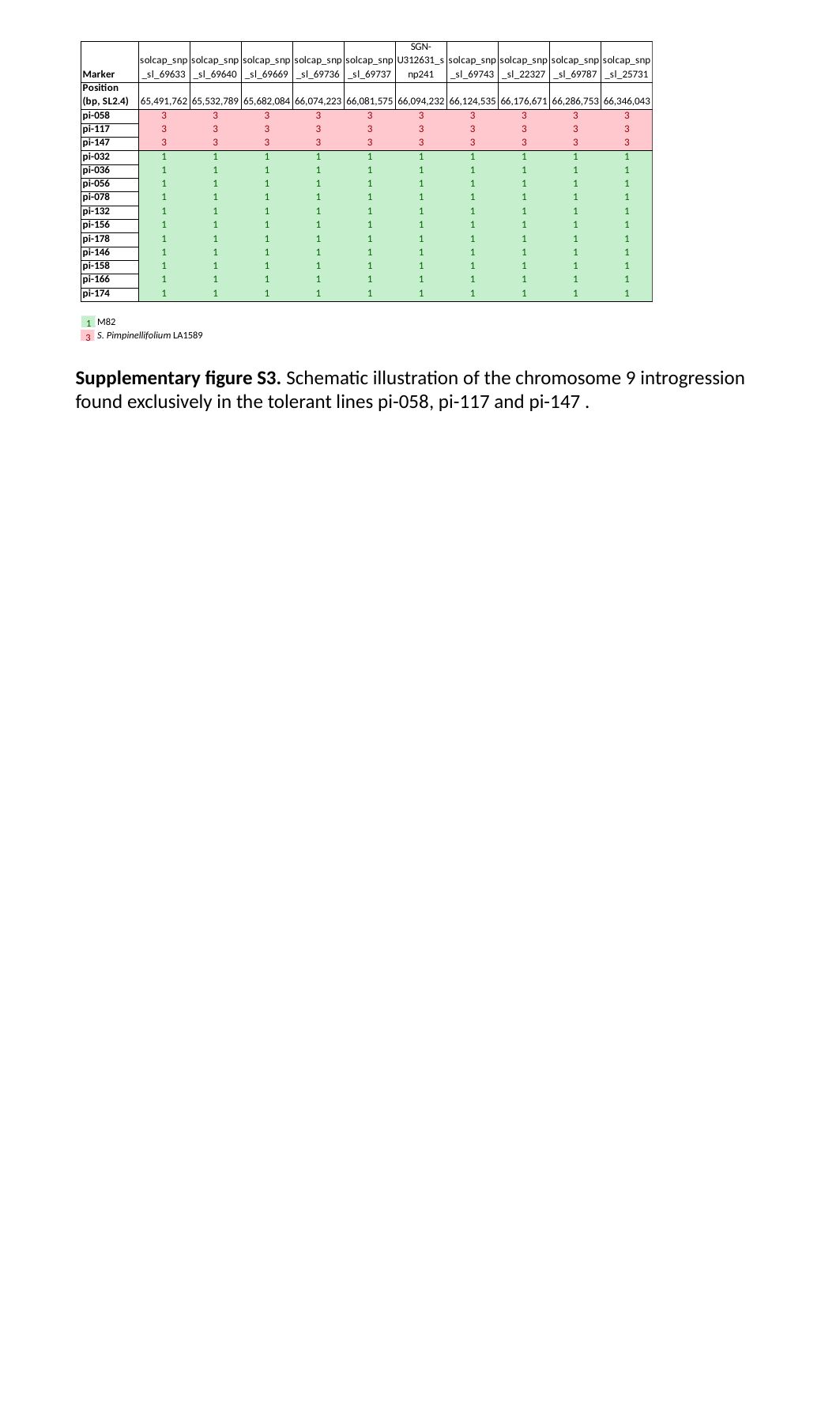

M82
1
S. Pimpinellifolium LA1589
3
Supplementary figure S3. Schematic illustration of the chromosome 9 introgression found exclusively in the tolerant lines pi-058, pi-117 and pi-147 .
