# Supplementary material for: New population of Solanum pimpinellifolium backcross inbred lines as a resource for heat stress tolerance in tomato
Source: Front Plant Sci. 2024 Jul 1;15:1386824. doi: 10.3389/fpls.2024.1386824 (PMC11246914; doi:10.3389/fpls.2024.1386824)
Supplement: Supplementary file 3 [file Table_1.docx]

Supplementary Material

# Supplementary Data

**Supplementary Table1.** List of annotated genes (SL2.4) in the common introgression on chromosome 9 of lines pi-058, pi-117 and pi-147.

| Chromosome | start | end | ID |
| --- | --- | --- | --- |
| SL2.40ch09 | 65,500,607 | 65,505,951 | Solyc09g090720.2 |
| SL2.40ch09 | 66,318,785 | 66,327,848 | Solyc09g091750.2 |
| SL2.40ch09 | 65,656,132 | 65,660,914 | Solyc09g090900.2 |
| SL2.40ch09 | 66,090,646 | 66,095,664 | Solyc09g091470.2 |
| SL2.40ch09 | 65,421,244 | 65,423,825 | Solyc09g090610.2 |
| SL2.40ch09 | 66,128,810 | 66,131,811 | Solyc09g091520.1 |
| SL2.40ch09 | 66,314,628 | 66,317,264 | Solyc09g091740.2 |
| SL2.40ch09 | 66,255,161 | 66,263,653 | Solyc09g091660.2 |
| SL2.40ch09 | 66,043,507 | 66,044,005 | Solyc09g091410.1 |
| SL2.40ch09 | 65,417,312 | 65,419,033 | Solyc09g090600.2 |
| SL2.40ch09 | 66,290,351 | 66,291,649 | Solyc09g091700.2 |
| SL2.40ch09 | 65,513,400 | 65,514,873 | Solyc09g090730.1 |
| SL2.40ch09 | 66,163,103 | 66,169,198 | Solyc09g091560.2 |
| SL2.40ch09 | 65,590,244 | 65,596,715 | Solyc09g090840.2 |
| SL2.40ch09 | 66,013,726 | 66,018,641 | Solyc09g091370.2 |
| SL2.40ch09 | 66,379,414 | 66,385,414 | Solyc09g091820.2 |
| SL2.40ch09 | 66,270,225 | 66,277,538 | Solyc09g091670.2 |
| SL2.40ch09 | 65,522,092 | 65,531,328 | Solyc09g090740.2 |
| SL2.40ch09 | 65,662,704 | 65,665,621 | Solyc09g090910.1 |
| SL2.40ch09 | 65,875,208 | 65,879,093 | Solyc09g091230.2 |
| SL2.40ch09 | 65,730,189 | 65,735,790 | Solyc09g091030.2 |
| SL2.40ch09 | 65,586,857 | 65,589,544 | Solyc09g090830.2 |
| SL2.40ch09 | 65,673,196 | 65,675,359 | Solyc09g090920.2 |
| SL2.40ch09 | 65,415,209 | 65,417,059 | Solyc09g090590.2 |
| SL2.40ch09 | 65,425,944 | 65,426,544 | Solyc09g090620.1 |
| SL2.40ch09 | 66,123,065 | 66,125,217 | Solyc09g091510.2 |
| SL2.40ch09 | 65,848,355 | 65,853,683 | Solyc09g091180.2 |
| SL2.40ch09 | 65,800,959 | 65,811,144 | Solyc09g091100.2 |
| SL2.40ch09 | 66,346,095 | 66,349,096 | Solyc09g091780.2 |
| SL2.40ch09 | 65,477,277 | 65,479,423 | Solyc09g090680.2 |
| SL2.40ch09 | 65,871,089 | 65,872,575 | Solyc09g091210.2 |
| SL2.40ch09 | 65,651,463 | 65,653,485 | Solyc09g090890.1 |
| SL2.40ch09 | 65,605,317 | 65,639,030 | Solyc09g090870.2 |
| SL2.40ch09 | 66,079,298 | 66,081,752 | Solyc09g091450.2 |
| SL2.40ch09 | 66,354,921 | 66,361,692 | Solyc09g091790.2 |
| SL2.40ch09 | 65,573,417 | 65,575,508 | Solyc09g090810.1 |
| SL2.40ch09 | 65,410,645 | 65,414,029 | Solyc09g090580.2 |
| SL2.40ch09 | 65,837,796 | 65,839,643 | Solyc09g091150.2 |
| SL2.40ch09 | 66,281,797 | 66,282,940 | Solyc09g091680.1 |
| SL2.40ch09 | 65,872,958 | 65,874,155 | Solyc09g091220.1 |
| SL2.40ch09 | 66,286,161 | 66,287,328 | Solyc09g091690.1 |
| SL2.40ch09 | 66,293,916 | 66,295,125 | Solyc09g091710.1 |
| SL2.40ch09 | 65,841,806 | 65,842,115 | Solyc09g091160.1 |
| SL2.40ch09 | 65,538,655 | 65,545,271 | Solyc09g090760.2 |
| SL2.40ch09 | 65,895,465 | 65,898,011 | Solyc09g091250.2 |
| SL2.40ch09 | 65,953,569 | 65,954,022 | Solyc09g091330.1 |
| SL2.40ch09 | 65,814,320 | 65,825,428 | Solyc09g091120.2 |
| SL2.40ch09 | 65,990,855 | 65,991,293 | Solyc09g091360.1 |
| SL2.40ch09 | 65,914,536 | 65,915,031 | Solyc09g091270.1 |
| SL2.40ch09 | 65,832,582 | 65,833,588 | Solyc09g091130.2 |
| SL2.40ch09 | 65,834,248 | 65,836,084 | Solyc09g091140.2 |
| SL2.40ch09 | 66,389,830 | 66,399,718 | Solyc09g091840.2 |
| SL2.40ch09 | 65,460,982 | 65,469,080 | Solyc09g090660.2 |
| SL2.40ch09 | 65,532,506 | 65,537,885 | Solyc09g090750.2 |
| SL2.40ch09 | 66,071,267 | 66,077,194 | Solyc09g091440.2 |
| SL2.40ch09 | 65,427,644 | 65,432,748 | Solyc09g090630.1 |
| SL2.40ch09 | 65,856,499 | 65,861,321 | Solyc09g091190.2 |
| SL2.40ch09 | 66,083,793 | 66,089,563 | Solyc09g091460.2 |
| SL2.40ch09 | 65,734,621 | 65,735,838 | Solyc09g091040.2 |
| SL2.40ch09 | 65,737,599 | 65,747,718 | Solyc09g091050.2 |
| SL2.40ch09 | 65,703,510 | 65,704,905 | Solyc09g090990.2 |
| SL2.40ch09 | 65,693,953 | 65,695,375 | Solyc09g090970.2 |
| SL2.40ch09 | 65,698,001 | 65,700,023 | Solyc09g090980.2 |
| SL2.40ch09 | 65,708,206 | 65,709,306 | Solyc09g091000.2 |
| SL2.40ch09 | 65,753,925 | 65,754,822 | Solyc09g091070.1 |
| SL2.40ch09 | 66,193,988 | 66,196,501 | Solyc09g091600.2 |
| SL2.40ch09 | 66,298,945 | 66,301,130 | Solyc09g091720.1 |
| SL2.40ch09 | 65,795,417 | 65,797,460 | Solyc09g091090.1 |
| SL2.40ch09 | 65,552,079 | 65,553,197 | Solyc09g090790.2 |
| SL2.40ch09 | 65,760,236 | 65,779,915 | Solyc09g091080.2 |
| SL2.40ch09 | 65,842,512 | 65,846,890 | Solyc09g091170.2 |
| SL2.40ch09 | 66,045,411 | 66,049,624 | Solyc09g091420.2 |
| SL2.40ch09 | 65,469,157 | 65,473,532 | Solyc09g090670.2 |
| SL2.40ch09 | 66,051,414 | 66,055,786 | Solyc09g091430.2 |
| SL2.40ch09 | 66,310,128 | 66,313,134 | Solyc09g091730.2 |
| SL2.40ch09 | 65,549,202 | 65,551,101 | Solyc09g090780.1 |
| SL2.40ch09 | 66,386,225 | 66,389,639 | Solyc09g091830.2 |
| SL2.40ch09 | 65,562,999 | 65,564,625 | Solyc09g090800.1 |
| SL2.40ch09 | 65,879,329 | 65,885,874 | Solyc09g091240.2 |
| SL2.40ch09 | 65,408,349 | 65,411,214 | Solyc09g090570.2 |
| SL2.40ch09 | 65,682,660 | 65,689,282 | Solyc09g090940.2 |
| SL2.40ch09 | 65,908,749 | 65,912,989 | Solyc09g091260.2 |
| SL2.40ch09 | 66,031,094 | 66,035,023 | Solyc09g091400.2 |
| SL2.40ch09 | 66,227,326 | 66,243,063 | Solyc09g091650.2 |
| SL2.40ch09 | 65,919,885 | 65,926,437 | Solyc09g091280.2 |
| SL2.40ch09 | 66,186,989 | 66,192,207 | Solyc09g091590.2 |
| SL2.40ch09 | 66,095,885 | 66,102,841 | Solyc09g091480.2 |
| SL2.40ch09 | 65,690,700 | 65,693,271 | Solyc09g090960.2 |
| SL2.40ch09 | 66,135,571 | 66,138,032 | Solyc09g091530.1 |
| SL2.40ch09 | 66,141,920 | 66,144,017 | Solyc09g091540.1 |
| SL2.40ch09 | 66,150,432 | 66,153,022 | Solyc09g091550.2 |
| SL2.40ch09 | 65,598,395 | 65,603,568 | Solyc09g090860.2 |
| SL2.40ch09 | 65,932,698 | 65,933,145 | Solyc09g091290.1 |
| SL2.40ch09 | 65,938,535 | 65,938,994 | Solyc09g091310.1 |
| SL2.40ch09 | 65,937,399 | 65,937,846 | Solyc09g091300.1 |
| SL2.40ch09 | 65,941,463 | 65,941,898 | Solyc09g091320.1 |
| SL2.40ch09 | 65,969,472 | 65,969,892 | Solyc09g091350.1 |
| SL2.40ch09 | 65,958,580 | 65,959,030 | Solyc09g091340.1 |
| SL2.40ch09 | 65,866,537 | 65,869,942 | Solyc09g091200.2 |
| SL2.40ch09 | 65,491,140 | 65,492,709 | Solyc09g090700.1 |
| SL2.40ch09 | 65,677,870 | 65,682,183 | Solyc09g090930.2 |
| SL2.40ch09 | 66,328,969 | 66,330,468 | Solyc09g091760.1 |
| SL2.40ch09 | 66,106,315 | 66,112,605 | Solyc09g091490.1 |
| SL2.40ch09 | 66,113,492 | 66,116,232 | Solyc09g091500.2 |
| SL2.40ch09 | 66,169,901 | 66,176,704 | Solyc09g091570.2 |
| SL2.40ch09 | 66,176,556 | 66,185,006 | Solyc09g091580.2 |
| SL2.40ch09 | 65,597,147 | 65,598,544 | Solyc09g090850.2 |
| SL2.40ch09 | 65,437,310 | 65,439,687 | Solyc09g090640.2 |
| SL2.40ch09 | 65,487,435 | 65,490,341 | Solyc09g090690.2 |
| SL2.40ch09 | 65,497,038 | 65,498,583 | Solyc09g090710.2 |
| SL2.40ch09 | 65,547,812 | 65,548,013 | Solyc09g090770.1 |
| SL2.40ch09 | 65,582,434 | 65,586,123 | Solyc09g090820.2 |
| SL2.40ch09 | 65,647,220 | 65,647,457 | Solyc09g090880.1 |
| SL2.40ch09 | 65,689,713 | 65,690,076 | Solyc09g090950.1 |
| SL2.40ch09 | 65,712,120 | 65,714,699 | Solyc09g091010.2 |
| SL2.40ch09 | 65,747,961 | 65,748,767 | Solyc09g091060.2 |
| SL2.40ch09 | 65,811,941 | 65,813,198 | Solyc09g091110.2 |
| SL2.40ch09 | 66,022,096 | 66,022,528 | Solyc09g091380.1 |
| SL2.40ch09 | 66,023,488 | 66,024,501 | Solyc09g091390.2 |
| SL2.40ch09 | 66,201,826 | 66,202,048 | Solyc09g091620.1 |
| SL2.40ch09 | 66,213,815 | 66,213,974 | Solyc09g091630.1 |
| SL2.40ch09 | 66,219,838 | 66,220,048 | Solyc09g091640.1 |
| SL2.40ch09 | 66,340,559 | 66,340,979 | Solyc09g091770.1 |
| SL2.40ch09 | 66,365,619 | 66,367,650 | Solyc09g091800.2 |
| SL2.40ch09 | 66,371,682 | 66,371,946 | Solyc09g091810.1 |
| SL2.40ch09 | 66,196,967 | 66,199,642 | Solyc09g091610.2 |
| SL2.40ch09 | 65,716,312 | 65,723,844 | Solyc09g091020.2 |
| SL2.40ch09 | 65,452,267 | 65,455,445 | Solyc09g090650.2 |
